# Effect of GABA, a Bacterial Metabolite, on *Pseudomonas fluorescens* Surface Properties and Cytotoxicity

**DOI:** 10.3390/ijms140612186

**Published:** 2013-06-06

**Authors:** Audrey Dagorn, Annelise Chapalain, Lily Mijouin, Mélanie Hillion, Cécile Duclairoir-Poc, Sylvie Chevalier, Laure Taupin, Nicole Orange, Marc G. J. Feuilloley

**Affiliations:** 1 Laboratory of Microbiology Signal and Microenvironment LMSM, EA 4312, Normandie University, Rouen University, GRRs SSE, IRIB, VASI, Evreux F-27000, France; E-Mails: audrey.dagorn@etu.univ-rouen.fr (A.D.); Annelise.Chapalain@iaf.inrs.ca (A.C.); lily.mijouin@gmail.com (L.M.); melanie.hillion@hotmail.fr (M.H.); cecile.poc@univ-rouen.fr (C.D.-P.); sylvie.chevalier@univ-rouen.fr (S.C.); nicole.orange@univ-rouen.fr (N.O.); 2 Laboratoire de Biotechnologie et Chimie Marines, Université de Bretagne-Sud B.P. 92116, Lorient cedex 56321, France; E-Mail: laure.taupin@univ-ubs.fr

**Keywords:** host-microbial interactions, cytotoxicity, virulence, bacterial adhesion, lipopolysaccharide, biofilms, pyoverdine

## Abstract

Different bacterial species and, particularly *Pseudomonas fluorescens*, can produce gamma-aminobutyric acid (GABA) and express GABA-binding proteins. In this study, we investigated the effect of GABA on the virulence and biofilm formation activity of different strains of *P. fluorescens*. Exposure of a psychotropic strain of *P. fluorescens* (MF37) to GABA (10^−5^ M) increased its necrotic-like activity on eukaryotic (glial) cells, but reduced its apoptotic effect. Conversely, muscimol and bicuculline, the selective agonist and antagonist of eukaryote GABA_A_ receptors, respectively, were ineffective. *P. fluorescens* MF37 did not produce biosurfactants, and its caseinase, esterase, amylase, hemolytic activity or pyoverdine productions were unchanged. In contrast, the effect of GABA was associated to rearrangements of the lipopolysaccharide (LPS) structure, particularly in the lipid A region. The surface hydrophobicity of MF37 was marginally modified, and GABA reduced its biofilm formation activity on PVC, but not on glass, although the initial adhesion was increased. Five other *P. fluorescens* strains were studied, and only one, MFP05, a strain isolated from human skin, showed structural differences of biofilm maturation after exposure to GABA. These results reveal that GABA can regulate the LPS structure and cytotoxicity of *P. fluorescens*, but that this property is specific to some strains.

## 1. Introduction

Gamma-aminobutyric acid (GABA) is a non-protein amino acid widespread in the environment [1]. Also, many bacteria, such as *Pseudomonas aeruginosa* [2,3], *Pseudomonas fluorescens* [4], marine *Pseudomonas* [5,6], lactic bacteria [7] and *Escherichia coli* [8], can synthesize GABA. Some of these bacteria release GABA, suggesting that GABA should act as a communication molecule between bacteria and their host or even between bacteria. Indeed, different GABA binding sites or sensors have been identified in bacteria. A GabP permease has been isolated in *E. coli* and *Bacillus subtilis* [9,10]. *Corynebacterium glutamicum* expresses a specific GABA transporter, also designated as GabP, but having low sequence identity with the GabP permease [11]. A selective GABA binding protein, Atu4243, was identified more recently in *Agrobacterium tumefaciens* and in several pathogenic or symbiotic Proteobacteria, including *Pseudomonas* [12]. Last year, we observed that in *P. aeruginosa*, GABA could bind the thermo-unstable ribosomal elongation factor, Tuf, a multiple function protein potentially translocated to the bacterial membrane, where it could act as a surface sensor [13]. A GABA binding protein (GBP), sharing common biochemical and pharmacological characteristics with the mammalian GABA_A_ receptor, was identified 20 years ago in an environmental strain of *P. fluorescens* [14], but this strain was patented and not deposited in international bacterial strains libraries. Until now, the recognition of GABA by other strains of *P. fluorescens* was not investigated, and the function of this molecule in *P. fluorescens* remains unknown.

In *Lactobacillus*, *Lactococcus lactis* and *E. coli*, GABA is involved in the resistance to acid stress [15,16], and in many species, including *Shigella flexneri* and *Listeria monocytogenes*, this system should be essential for colonization of the gastro-intestinal tract [17]. The physiological role of GABA was investigated in detail in *A. tumefaciens*, where it was shown that this molecule, which is released by plants in response to infection, induces the expression of a lactonase that cleaves *N*-3-oxo-octanoyl-l-homoserine lactone (3-oxo-C8-HSL), leading to a reduction of tumor formation [18]. GABA is also an inhibitor of virulence in the phytopathogen *Pseudomonas syringae* pv. tomato, where at high concentrations, it downregulates the expression of *hrp* genes and increases the resistance of the plant to infection [19]. Conversely, in the human opportunistic pathogen, *P. aeruginosa*, GABA reduces biofilm formation activity, but increases the production of hydrogen cyanide and its virulence [13]. It was then particularly interesting to investigate the effect of GABA on *P. fluorescens*, which is frequently found in the rhizosphere, where it can enhance the resistance of plants against bacterial pathogens, including *P. syringae* pv. tomato [20], but is also found in the clinical environment, where some strains adapted to the human physiological temperature behave clearly as opportunistic pathogens [21].

In the present study, we investigated the effect of GABA on the growth kinetic, mobility, cytotoxic activity, binding potential on biotic and abiotic surfaces, biofilm formation activity, surface polarity, biosurfactant production, lipopolysaccharide (LPS) structure, exoenzymes secretion and pyoverdine production by environmental and clinical strains of *P. fluorescens*.

## 2. Results

### 2.1. Effect of GABA on the Cytotoxic Activity, Adhesion Potential, Biofilm Formation Activity and Surface Properties of *Pseudomonas fluorescens*

**Figure 1:**
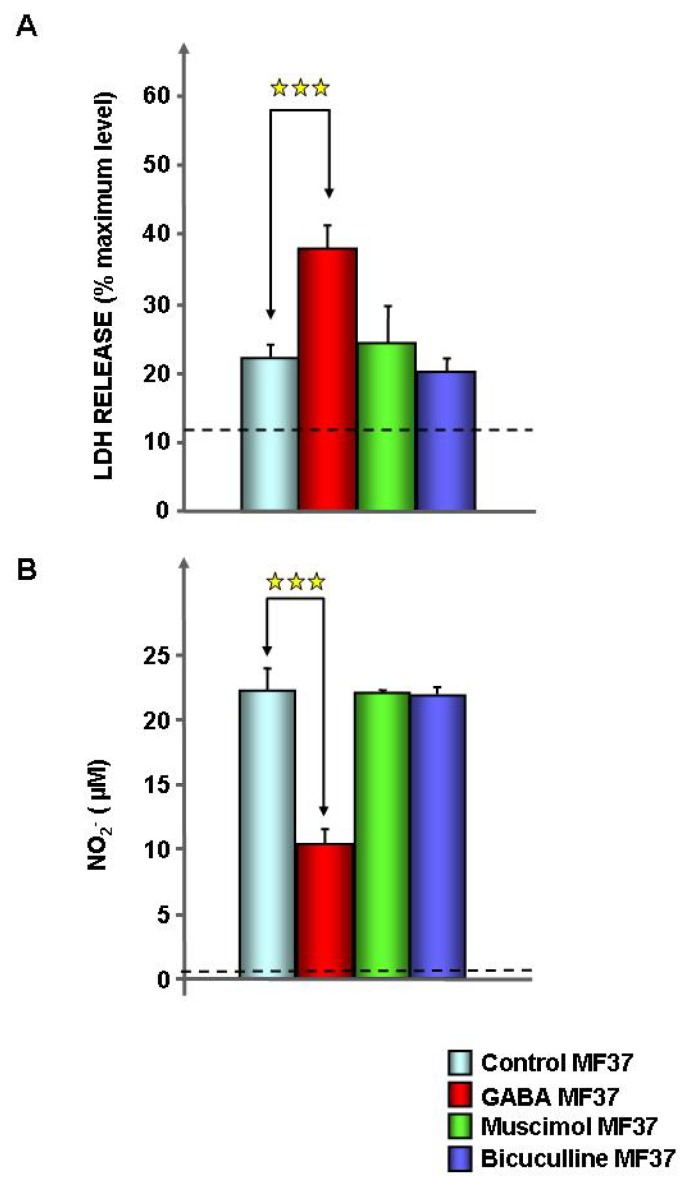
Effect of GABA, the GABA_A_ receptor agonist muscimol and the GABA_A_ receptor antagonist bicuculline (10^−5^ M) on the cytotoxicity of *P. fluorescens* MF37 on eukaryotic (glial) cells. The necrotic activity of the bacterium was determined by assay of lactate dehydrogenase (LDH) in the culture medium. (**A**) The apoptotic activity was evaluated by the assay of nitrite; (**B**) in which nitrites are produced upon activation of NO-synthase in the target cell. Dotted lines indicate the production of LDH and nitrites in the medium of control eukaryotic cells not exposed to the bacterium (★★★: *p* < 0.001).

Before the tests, bacteria were exposed to GABA 10^−5^ M during the whole growth phase and rinsed to remove any trace of free GABA. The concentration of 10^−5^ M was selected to avoid any possible metabolic or pH effect and because it is the mean value of the concentrations in the synaptic cleft of neurons (1.5–3 mM [22]), in wounded vegetal (10^−4^ M [23]) and in the environment (sea water concentration <10^−7^ M [24]). Administration of GABA did not modify the growth kinetic or the swimming and swarming mobility of the psychrotrophic strain of *P. fluorescens* MF37 (data not shown). The cytotoxic activity of *P. fluorescens* MF37 was studied using a model of cultured rat glial cells [25], and the cell viability was determined by measurement of the release of lactate dehydrogenase (LDH) and nitrite ions in the culture medium. In comparison to the cells exposed to control bacteria, when glial cells were exposed to *P. fluorescens* MF37 treated with GABA (10^−5^ M), we observed a significant increase of LDH release (+71.5% ± 5.4%, *p* < 0.001) (Figure 1A) and a decrease of the nitrite ion concentration (−53.3% ± 2.9%, *p* < 0.001) (Figure 1B). Bacteria exposed to GABA 10^−6^ M also showed an increase of cytotoxicity, but this effect was not significant. Muscimol (10^−5^ M), a selective agonist of eukaryote GABA_A_ receptors, and bicuculline (10^−5^ M), an antagonist of these receptors, were without effect on *P. fluorescens* MF37 cytotoxicity. Because glial cells can also detect GABA [26], control studies were realized by direct treatment of glial cells with GABA (10^−5^ M), and we observed that GABA had no effect on glial cell viability. As previously demonstrated [25,27], in our experimental conditions, *P. fluorescens* MF37 did not release detectable amounts of LDH and NO.

**Figure 2:**
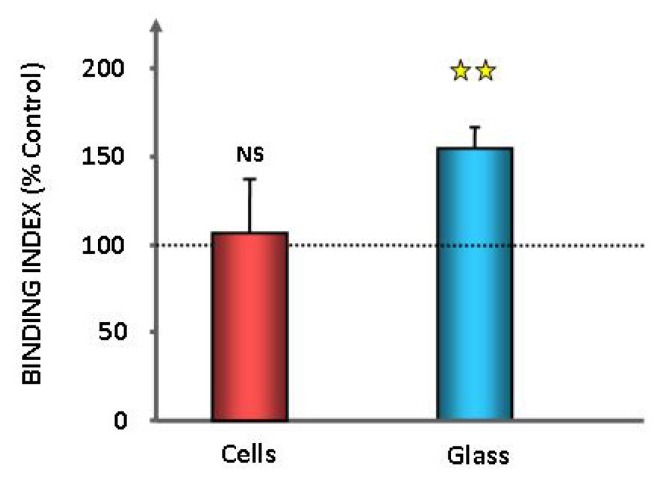
Effect of GABA (10^−5^ M) on the adhesion properties of *P. fluorescens* MF37 to eukaryotic cells or glass. Adhesion is expressed as the percentage of the control value (dotted line) (NS: non-significant; ★★: *p* < 0.01).

**Figure 3:**
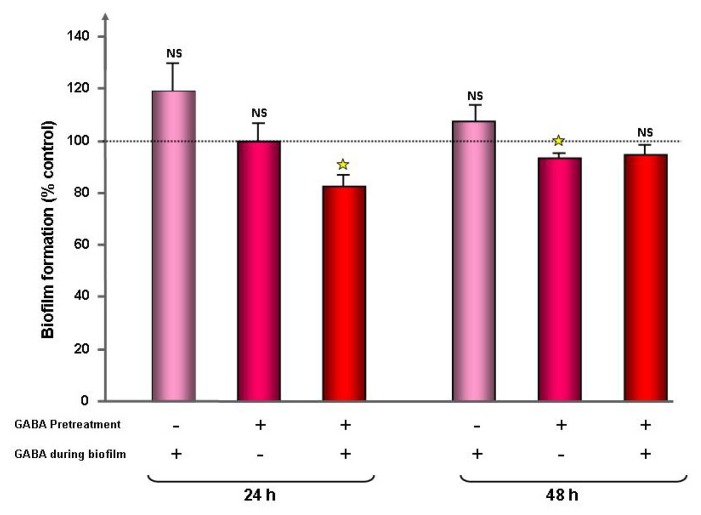
Effect of GABA (10^−5^ M) on the biofilm formation activity of *P. fluorescens* MF37 in 96-well PVC microtitration plates. The biofilm formation activity of bacteria was measured after 24 or 48 h of incubation. Bacteria were exposed to GABA only during the growth phase (pretreatment), only during the biofilm formation period (during biofilm) or continuously during growth and biofilm formation (pretreated + during biofilm). The biofilm formation activity is expressed as the percentage of the control value (dotted line). (NS: non-significant; ★: *p* < 0.05).

**Figure 4:**
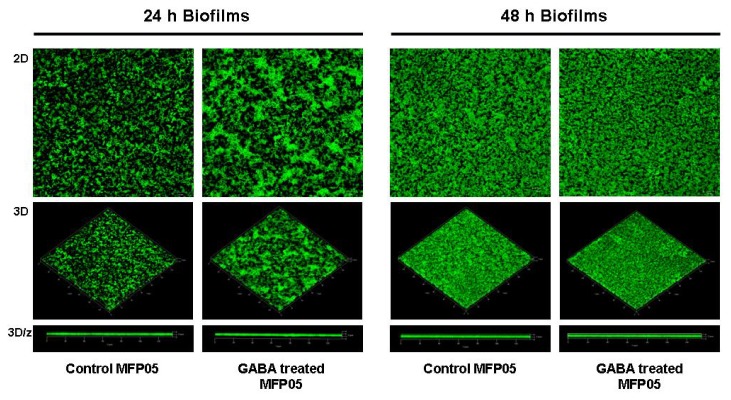
Effect of GABA (10^−5^ M) on the biofilm formation activity of *P. fluorescens* MFP05 on glass slides. 2D, 3D and 3D/z figures resulting from confocal laser scanning observations were realized after 24 and 48 h of biofilm formation.

As adhesion is a key step in the expression of bacterial virulence, we investigated the adhesion potential of GABA treated *P. fluorescens* MF37 on cultured glial cells and glass. The binding index of GABA treated bacteria on primary cultures of rat glial cells was unchanged, but we observed a significant increase of adhesion (+55% ± 12%, *p* < 0.01) on glass surfaces (Figure 2). The effect of GABA on *P. fluorescens* MF37 biofilm formation was studied using the crystal violet technique and confocal microscopy. When *P. fluorescens* MF37 was pre-treated only during the exponential growth phase, the crystal violet technique revealed a limited, but significant reduction of biofilm formation on polyvinyl chloride (PVC) microtitration plates (−7% ± 2%, *p <* 0.05) after 48 h incubation, whereas there was no difference after 24 h (Figure 3). Conversely, when the bacteria were pre-treated during the exponential growth phase and the biofilm formation period, we observed a significant reduction of the biofilm after 24 h (−18% ± 4%, *p <* 0.05), but this effect was not maintained at 48 h. A unique treatment with GABA during the biofilm formation period was without effect. When the biofilm formation activity of *P. fluorescens* MF37 was studied on glass slides by confocal microscopy, we did not observe any change in the morphology or thickness of 5, 24 and 48 h-old biofilms. Other strains of *P. fluorescens* originating either from agricultural soil (Pf0-1), plant leaves (SBW25), human skin (MFP05) or from the pulmonary tract and considered as an opportunistic pathogen (MFN1032) were studied in the same conditions. Only the biofilm formed by MFP05 was affected by GABA (Figure 4). There was no difference between the biofilms formed by control and GABA-treated bacteria after 5 h incubation. In contrast, the 24 h-old biofilm of GABA-treated MFP05 showed an irregular bacterial cluster and appeared more mature, its aspect being identical to a 30 h-old biofilm. This effect was transient, and after 48 h, the aspect of control and GABA-treated *P. fluorescens* MFP05 biofilms was the same.

**Figure 5:**
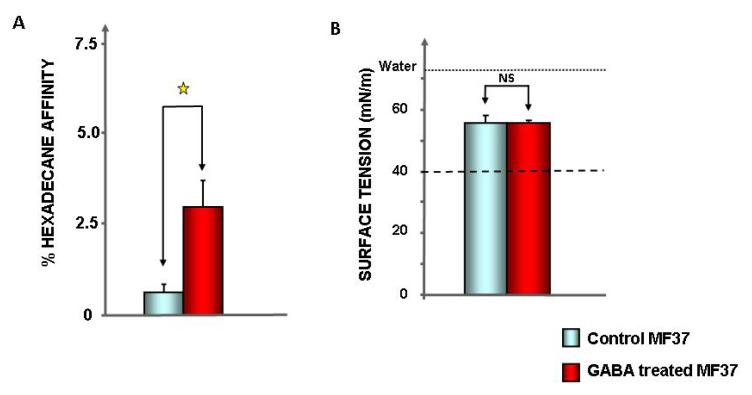
Effect of GABA (10^−5^ M) on the global surface polarity (**A**) and biosurfactant production (**B**) of *P. fluorescens* MF37. The surface polarity was determined by measurement of the affinity of bacteria to hexadecane. The biosurfactant production was estimated by measurement of the surface tension of rinsing solutions of bacterial colonies grown on solid agar medium. (NS: non-significant; ★: *p* < 0.05).

As these results suggested an evolution of the surface properties of *P. fluorescens*, we investigated, by the microbial adhesion to solvent (MATS) technique, the surface hydrophilic character of the microorganism. The affinity of *P. fluorescens* MF37 to hexadecane was significantly increased after treatment by GABA (0.6% ± 0.2% for control, 3% ± 0.7% for GABA-treated bacteria, *p* < 0.05) (Figure 5A), but the affinity of MF37 to hexadecane remained below 20%, which is considered the threshold value for highly hydrophilic bacteria [28]. Then, it appears that the global polarity of this strain was unchanged. Complementary studies showed that the surface tension from the rinsing medium of *P. fluorescens* MF37 colonies was also the same before or after GABA treatment (55.7% ± 0.4 and 55.8 ± 2.2 mN/m, respectively) (Figure 5B), indicating that in both conditions, *P. fluorescens* MF37 was not producing biosurfactant susceptible to modulation of bacterial adhesion.

### 2.2. Effect of GABA on Secreted Diffusible Factors and Lipopolysaccharide Structure

**Figure 6:**
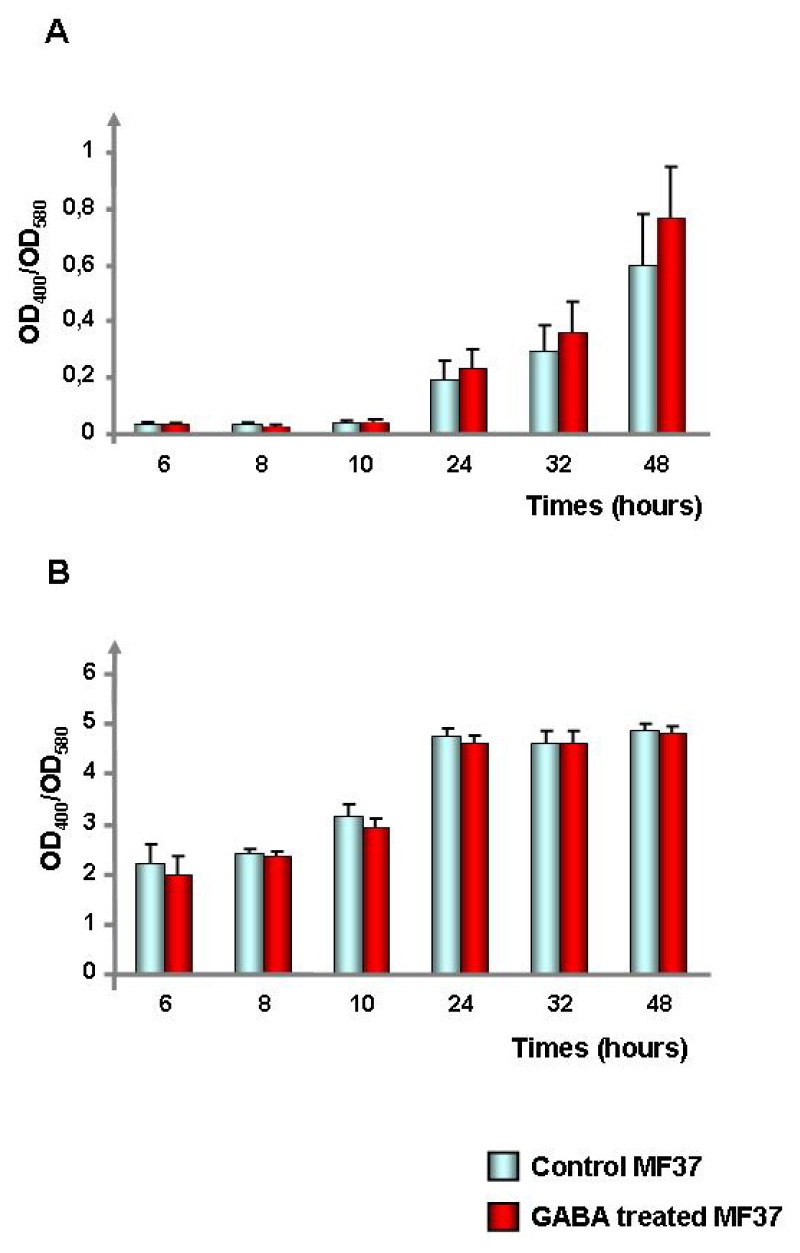
Effect of GABA (10^−5^ M) on pyoverdine production by *P. fluorescens* MF37. The pyoverdine production was measured in King B (**A**) and Bacto Casamino Acids (CAA) (**B**) medium. The production of pyoverdine is expressed as the ratio of pyoverdine adsorption (OD_400_) on the OD_580_ nm of the bacterial culture.

In order to identify the origin of the effect of GABA on *P. fluorescens* MF37 cytotoxicity, we investigated the effect of GABA on the caseinase, esterase, amylase, elastase and hemolytic secreted activities of the bacterium, but no difference was observed between control and GABA-treated bacteria (data not shown). Then, we investigated the effect of GABA on the production of pyoverdine, a siderophore essential for the colonizing activity of *Pseudomonas* and for virulence in plants and animals [29]. Using King B medium, a time-dependent increase of pyoverdine production was observed with both control and GABA-treated bacteria (Figure 6A). After 24, 32 and 48 h of incubation, the OD_400_/OD_580_ ratio used to monitor pyoverdine production was higher in GABA-treated bacteria, but the difference with control bacteria was not significant. For that reason, the same experiment was repeated using Bacto Casamino Acids (CAA) medium, another pyoverdine-inducing medium. An increase of pyoverdine was observed from 6 to 48 h of incubation, but it appeared clearly that GABA was not modulating pyoverdine production (Figure 6B).

**Figure 7:**
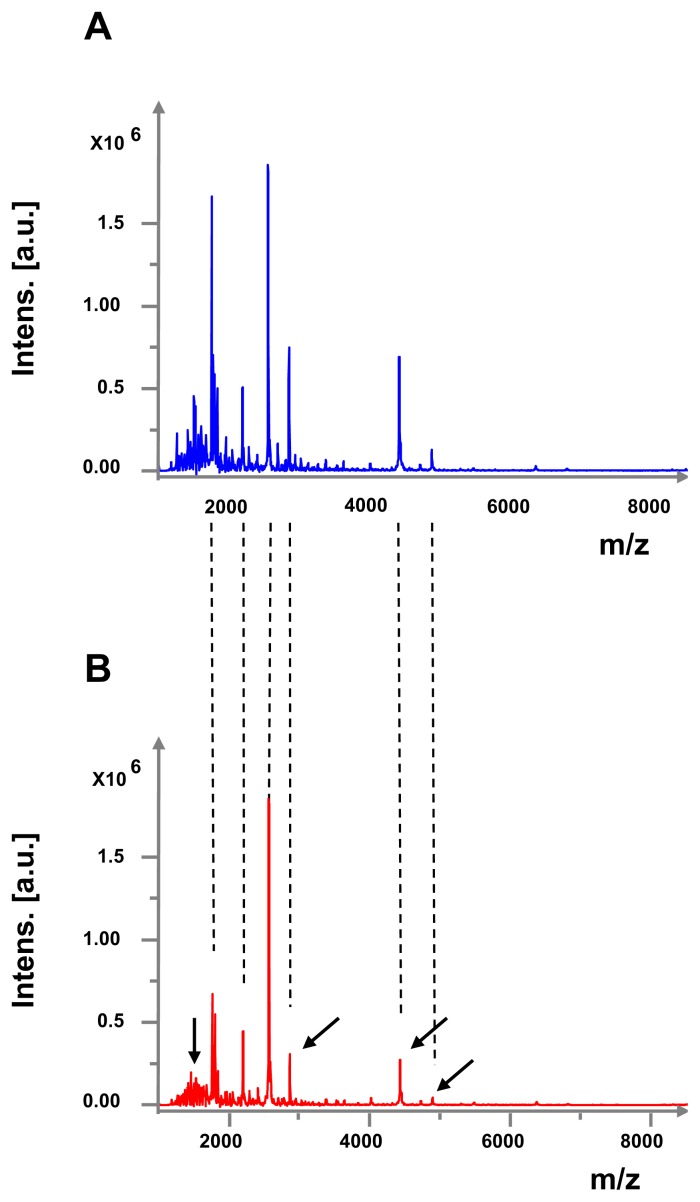
MALDI-TOF analysis of the lipopolysaccharide (LPS) extracted from control (**A**) and GABA-treated (**B**) *P. fluorescens* MF37. Each figure is a representative example of three different analysis. Arrows indicate peaks particularly modified between control and GABA-treated bacteria.

The lipopolysaccharide (LPS) is one of the major virulence factors and the principal endotoxin of *Pseudomonas*. LPS extracted from *P. fluorescens* MF37 exposed or not to GABA was studied by MALDI-TOF mass spectrometry. We observed that the mass spectra of LPS extracted from control bacteria (Figure 7A) showed limited, but significant and repeatable, differences in comparison with the mass spectra of GABA-treated bacteria (Figure 7B). The modified peaks were found essentially in the region of low m/z fragments, considered as corresponding to lipid A fragments [30]. Three peaks of a higher m/z ratio, presumably associated with fragments of the oligosaccharide core [30], were also particularly decreased in GABA-treated bacteria, suggesting that GABA induced a large rearrangement of the LPS structure.

## 3. Discussion

GABA is present from eukaryotes to bacteria and should have a pivotal role in inter-kingdom communication [31,32]. We have recently described that GABA stimulates the virulence of the opportunistic pathogen, *P. aeruginosa* [13]. The existence of GABA binding proteins or sensors in other *Pseudomonas* and, particularly, in *P. fluorescens* has been known for a long time [14], but the physiological effect of GABA in this species was not investigated until now.

As shown with *P. aeruginosa* PAO1, GABA (10^−5^ M) had no effect on the growth kinetic and mobility of *P. fluorescens* MF37, but markedly affected the cytotoxic activity of this bacterium. LDH is a stable eukaryotic enzyme considered as a marker of necrosis or non-specific cell death, whereas nitrite ions can be employed as markers of apoptosis, since they are generated by the spontaneous conversion of nitric oxide (NO) during eukaryotic cells apoptosis [25,33]. Whereas the release of LDH from glial cells exposed to GABA-treated bacteria was increased, the concentration of NO was significantly decreased. These results are only contradictory in appearance, since the time course of necrosis is more rapid than required for induction of apoptosis [34], and a rapid induction of necrosis can prevent the activation of apoptosis [35]. It is also interesting to note that the absence of response of *P. fluorescens* MF37 to muscimol and bicuculline suggests that in this strain, the GABA sensor is not related to the muscimol-sensitive GBP protein identified by Guthrie *et al.* [36] in an environmental strain of *P. fluorescens*. The virulence of *P. fluorescens* is highly variable and appears strain- and environment-dependent. A psychrotrophic strain, such as *P. fluorescens* MF37, has a marked cytotoxic activity on animal cells [25], and some clinical strains have an infectious potential in the same range as *P. aeruginosa* [21]. Conversely, in plants, *P. fluorescens* is essentially, if not always, considered as a protecting agent [20,37]. Also, GABA appears to have opposite effects on bacterial virulence in plants and animal models. Indeed, as in *P. aeruginosa* [13], GABA acts as promoter of *P. fluorescens* cytotoxicity on rat glial cells, whereas in a phytopathogen, such as *A. tumefaciens*, GABA reduces the virulence [18]. This should be correlated to the different functions of GABA in plants, where it is a main contributor of the defense system [38], and in animals, where its protective role is apparently marginal [39]. Alternatively, these differences should reflect opposite adaptation processes between α- and γ-Proteobacteria, such as *Agrobacterium* and *Pseudomonas*.

Bacteria have two lifestyles, *i.e.*, planktonic and in biofilms. In biofilms, bacteria are protected against the action of antibiotics and host immune system molecules [40]. We tested the effect of GABA on *P. fluorescens* adhesion and biofilm formation activity on living cells, PVC (hydrophobic) and glass (hydrophilic) surfaces. GABA did not modify the initial binding index of *P. fluorescens* MF37 on living glial cells, but increased its adhesion on glass. On PVC, we observed a significant reduction of the biofilm formation activity only after 48 h, when the bacteria were pretreated with GABA during their growth phase, and after 24 h (but not after 48 h), when the bacteria were pretreated both during the growth phase and the biofilm formation period. These results suggest that only bacteria in the early growth phase are sensible to GABA either, because they express a sensor differently or, as in biofilm, GABA is unable to affect their physiology. We also tested the effect of GABA on the biofilm formation activity of bacteria on glass to observe the biofilm structure. This study was performed on *P. fluorescens* MF37, but also in other strains from environmental (Pf0-1, SBW25), clinical (MFN1032) or human skin (MFP05) origin. GABA did not affect the structure and the thickness of the biofilms, except in the case of MFP05, where GABA induced an apparent increase of the biofilm maturation speed. GABA is synthesized and released in skin [41], and the response of MFP05 to GABA should represent an adaptation response to host signals. It is interesting to note that human skin *P. fluorescens* are special. Indeed, whereas these bacteria are found in abundance on skin regions in metagenomic studies [42], they are rarely cultivable [43], suggesting that they require specific growth conditions. In agreement with our results, this points out the high heterogeneity of *P. fluorescens* and their dependence in regards to the microenvironment.

As bacterial adhesion is governed by the surface properties, we investigated the effect of GABA on the hydrophobicity, biosurfactant production and LPS structure of *P. fluorescens* MF37. The surface hydrophobicity of the bacteria was estimated by the MATS technique. The affinity of *P. fluorescens* MF37 to hexadecane is under 20% ; then, according to Bellon-Fontaine *et al.* [28], this strain was hydrophilic in our experimental conditions. GABA significantly increased the affinity of *P. fluorescens* MF37 to hexadecane, but the general hydrophilic character of the bacterium was preserved. It is known that biosurfactants synthesized by *P. fluorescens* are implicated in surface adhesion and biofilm formation [44]. The possible effect of GABA on biosurfactants production was studied by direct measurement of the surface tension of the rinsing medium of bacterial colonies. In control medium, this value was over 40 mN/m, indicating that *P. fluorescens* MF37 was not producing biosurfactants, and this surface tension was unchanged after GABA treatment. As in parallel, GABA did not interfere with the swimming or swarming potential of *P. fluorescens* MF37; this suggests that the structure and activity of flagella and pili were not modified. This is not excluding that GABA could interfere with other adhesins, but, if any, this effect should be marginal.

*P. fluorescens* releases different diffusible enzymatic activities, but none was modified by GABA. Similarly, pyoverdine, which can trigger cytotoxicity [45], was also unaffected. In order to identify the factor responsible for the evolution of the cytotoxicity and surface properties of GABA-treated *P. fluorescens* MF37, we investigated the effect of GABA on the LPS structure. LPS is the major endotoxin of *Pseudomonas*, and it is known to play a key role in bacterial adhesion [46,47]. MALDI-TOF mass spectra of LPS extracted from control and GABA-treated *P. fluorescens* MF37 showed limited, but repeatable differences. These differences were essentially observed in low m/z LPS sub-fragments that should be generated by cleavage of lipid A. Interestingly, in *P. aeruginosa*, structural changes in this region have been associated with an increase in necrotic activity [30]. As observed in the case of growth temperature variations [27] or exposure to natriuretic peptides [35], the virulence of *P. fluorescens* MF37 appears modulated by a rearrangement of the LPS structure. It should be noted that NO has, by itself, a high cytotoxicity on glial cells [48], but its production by cells exposed to GABA-treated bacteria was reduced, suggesting that its contribution to glial cell death is marginal. Then, although *P. fluorescens* MF37 and *P. aeruginosa* PAO1 respond to GABA by an increase in cytotoxicity, the mechanisms appear to be very different. In *P. aeruginosa*, the effect of GABA is due to an over-production of a diffusible virulence factor (HCN) [13], whereas in *P. fluorescens*, it is essentially, if not only, dependent on the LPS structure and, then, contact-dependent.

## 4. Experimental Section

### 4.1. Bacterial Strains and Culture Conditions

*P. fluorescens* MF37 is a natural rifampicin-resistant mutant of the strain MF0 from raw milk [49]. This strain is a model of psychrotrophic *Pseudomonas* widely used in our laboratory. *P. fluorescens* MFN1032 is a clinical strain adapted to grow at 37 °C collected from a pulmonary tract infection [21]. It is considered to be a nosocomial bacterium. *P. fluorescens* MFP05 was collected from human skin, where this species is considered as a member of the transient bacterial microflora. *P. fluorescens* SBW25 and Pf0-1 are environmental bacteria. The SBW25 strain was isolated from plant leaves, whereas the Pf0-1 strain was isolated from agricultural soil [50]. For pre-treatment, bacteria were transferred to 25 mL of nutrient broth (NB, Merck, Darmstadt, Germany) containing or not containing GABA (10^−5^ M) and were cultured at 28 °C, until the beginning of the stationary phase. The density of the bacterial suspension was determined by absorption at 580 nm (ThermoSpectronics, Cambridge, UK).

### 4.2. Swimming and Swarming Mobility Tests

Cultures of *P. fluorescens* MF37 in NB in the early stationary phase were collected and centrifuged (10 min, 6000 rpm). For the swimming mobility test, plates containing 0.3% agar were inoculated on the surface using a needle previously soaked with the centrifugation pellet. The plates were incubated at 28 °C until the development of a migration halo. The diameter of the halo was measured between 4 and 48 h after inoculation. The values of mobility were determined over a minimum of 3 independent measures. For the swarming mobility test, the same protocol was used, except that NB medium was supplemented with 0.5% agar.

### 4.3. Cytotoxic Activity Tests

The effect of GABA, muscimol and bicuculline on the cytotoxic activity of *P. fluorescens* MF37 was investigated on primary cultures of rat glial cells using biochemical indicators of apoptosis and necrosis, as previously described [25,27]. Rat glial cells, obtained from newborn (24–48 h) rat brain, were grown in DMEM/Ham’s medium (2/1) supplemented with 10% fetal calf serum, 2 mM glutamine, 0.001% insulin, 5 mM HEPES, 0.3% glucose and 1% antibiotic-antimycotic solution (Biowhittaker, Emerainville, France). The cells were layered at a concentration of 10^5^ cells/well on 24 well-plates coated with poly-l-lysine (50 μg.mL^−1^) and kept at 37 °C in a 5% CO_2_ humidified atmosphere. Glial cells were allowed to grow for 12–16 days before use. For the tests, bacteria were pretreated with GABA overnight (18 h) at 28 °C. Before the tests, bacteria in the stationary phase were harvested by centrifugation (6000 rpm, 5 min) and rinsed 3 times to remove any trace of free GABA. Bacteria were then re-suspended at a density of 10^6^ CFU.mL^−1^ in glial cell culture medium without antibiotics and antimycotics and incubated with glial cells during 24 h. The concentration of LDH was determined using the Cytotox 96^®^ Enzymatic Assay (Promega, Charbonnieres, France). Nitrite ions (NO_2_^−^) resulting from the spontaneous conversion of nitric oxide (NO) in the incubation medium were measured using the Griess colorimetric reaction.

### 4.4. Evaluation of the Bacterial Biofilm Formation Activity by the Crystal Violet Technique

The biofilm formation activity of *P. fluorescens* MF37 was studied using the crystal violet technique adapted from O’Toole and Kolter [51]. Cultures of *P. fluorescens* MF37 grown in NB and in the early stationary phase were centrifuged for 10 min at 6000 rpm and rinsed with sterile water NaCl 0.9% (SPW). Then, an aliquot of 100 μL of a bacterial culture adjusted to an OD_580_ = 0.4 was layered in a polystyrene microtitration plate (96 wells) and incubated for 24 or 48 h. In a first series of experiments, bacteria were grown in the absence of GABA, but were exposed to the molecule during the biofilm formation. In a second series of experiments, bacteria were grown in the presence of GABA, but GABA was not added to the medium during the biofilm formation step. In a third series of experiments, bacteria were grown with GABA, as previously, but the medium in PVC microtitration plates was supplemented with GABA to maintain the treatment during the 24 to 48 h of biofilm formation. At the end of the incubation time (24 h or 48 h) at 28 °C, the OD_595_ was measured. After removal of the bacterial suspension and rinsing with SPW, bacteria and matrix bound to the wells were stained with 150 μL of crystal violet (0.1%, 30 min). After rinsing, the dye was recovered by adding 150 μL of SDS (1% in sterile water, 15 min), and the OD_595_ nm of the solution was measured. Data were normalized as a percentage of biofilm density in the absence of treatment. The percentage of biofilm formation was evaluated by the following equation:

(1) $$\%\;\text{of biofilm formation}=({\text{OD}}_{595\;\text{after crystal violet staining}}/{\text{OD}}_{595\;\text{before crystal violet staining}})\times 100$$

### 4.5. GFP Transformation

*P. fluorescens* MF37, SBW25, MFN1032, Pf0-1 and MFP05 were transformed with the pSMC2.1 plasmid [52]. The plasmid was extracted from an *E. coli* strain containing the green fluorescent protein (GFP) gene and a kanamycin resistance cassette. Briefly, 1.5 mL aliquots of bacterial cultures realized in LB medium at 28 °C and reaching an OD_580_ between 1 and 2 were centrifuged for 1 min at 13,000 rpm. The pellet was washed 3 times with 300 μL of sucrose (300 mM) and re-suspended in 100 μL of sucrose (300 mM). For the Pf0-1 strain, the pellet was washed and re-suspended using cold distilled water. Competent cells were mixed with 5 μL of pSMC2.1 plasmid and electroporated at 1800 V. After electroporation, bacteria were incubated in 1 mL of LB medium for 1 h at 28 °C under 180 rpm agitation. A small volume of the culture (100 μL) was layered on LB agar supplemented with kanamycin (200 μg/mL). After 24 h of incubation, transformed bacteria were observed by confocal microscopy (LSM 710, Zeiss, Oberkochen, Germany) to verify the integration of the plasmid and the production of a green fluorescent signal.

### 4.6. Confocal Microscope Study of the Bacterial Biofilm Formation Activity on Glass Surfaces

A pre-culture and cultures of the GFP transformed strains of *P. fluorescens* were realized in NB medium, supplemented with kanamycin (200 μg/mL) for MFN1032, MF37 and SBW25. The plasmid was stable in the strains MFP05 and Pf0-1, and the antibiotic pressure was not necessary. After 24 h of incubation with or without GABA (10^−5^ M), bacteria were centrifuged at 7000× *g* for 10 min at room temperature. The pellets were washed one time with SPW and adjusted at OD_580_ = 1 in a final volume of 25 mL. Glass slides, washed beforehand with ethanol, were placed in Petri dishes, covered with the bacterial suspension and incubated at 28 °C in static condition. After 2 h, glass slides were washed two times with SPW. One slide was heath fixed to control the adhesion capacity of the bacteria, and the others were covered with 25 mL of NB and incubated at 28 °C. After 5, 24 and 48 h of incubation, one glass slide was washed two times with SPW, dried, fixed and observed with a confocal microscope (LSM 710, Zeiss) using an ×63 immersion objective. Images were processed using Zen software (Zeiss, 2009); a median filter was applied, images were segmented and fluorescence was quantified.

### 4.7. Evaluation of Bacterial Surface Properties

The binding index of bacteria on biological (cell) surfaces was determined using the gentamicin exclusion test [33]. Glial cells were exposed to bacteria (10^6^ CFU·mL^−1^) in culture medium without antibiotics and antimycotics for 4 h. At the end of the incubation period, cultures were rinsed 3 times with fresh medium to remove unattached bacteria. A part of the wells was immediately treated with 500 μL of Triton X100 in SPW (0.1% *v*/*v*) for 15 min at 37 °C. After plating and incubation for 2 days at 28 °C, the total number of bacteria present at the surface and in the cells was determined. Other wells were exposed to gentamicin (300 μg·mL^−1^) for 1 h at 37 °C, rinsed 3 times and then treated with Triton X100 (0.1%) for 15 min. Extracellular bacteria were killed by gentamicin, and the colonies obtained after culture correspond to bacteria exclusively present in the intracellular compartment. The number of adherent bacteria was calculated as the difference between the total number and the intracellular bacteria.

The binding index of bacteria on glass slides was determined by direct counting. Before use, glass slides were cleaned by immersion in ethanol (70% in water), rinsed in sterile water and treated with TFD4 detergent (4% in 50 °C water) for 1 h to remove any traces of lipids. Then, glass slides were rinsed in sterile water and dried under laminar air flow. A bacterial suspension (10^8^ CFU·mL^−1^ in SPW) was layered on each glass slide. Bacteria were allowed to adhere for 2 h at 28 °C. Non-adherent bacteria were removed by rinsing with SPW, and the remaining adherent bacteria were immediately stained with acridine orange (0.01% in SPW) for 20 min. After rinsing in SPW and drying, the slides were observed using an epifluorescence microscope Zeiss Axiovert 100 equipped with a Nikon DXM1200F color camera. The binding index was determined by counting a minimum of 20 homologous fields.

The surface polarity of bacteria was determined using the microbial adhesion to solvent (MATS) technique [28]. Bacterial cultures treated or not with GABA (10^−5^ M) were harvested by centrifugation for 10 min at 10,000× *g*. Pellets were rinsed 2 times with SPW and diluted to OD_400_ = 0.8. An aliquot of bacterial suspension (2.4 mL) was mixed with 0.4 mL of hexadecane in glass tubes. The tubes were vigorously hand-shaken for 10 s, vortexed for 45 s and hand-shaken again for 10 s. After 15 min, the OD_400_ of the aqueous phase was measured. The percentage of affinity for hexadecane was calculated by the relation: [(OD control − OD test)/OD control] × 100.

In this relation, OD control corresponds to the OD_400_ of bacterial suspension without hexadecane, and OD test corresponds to the OD_400_ of bacterial suspension with hexadecane.

### 4.8. Surface Tension and Biosurfactant Production in Bacterial Culture Medium

The biosurfactant production was monitored indirectly by measuring the surface tension of the rinsing solution (SPW) of colonies of *P. fluorescens* grown during 48 h on solid NB medium, using the Wilhelmy plate technique [53].

### 4.9. Study of the Lipopolysaccharide Structure

The lipopolysaccharide (LPS) was purified from *P. fluorescens* MF37, as described by Darveau and Hancock [54]. Bacteria in the early stationary phase were harvested by centrifugation (6000× *g*, 10 min, 4 °C). Each pellet was re-suspended in 10 mM Tris-buffer 10 mM containing 2 mM MgCl_2_, 200 μg mL^−1^ pancreatic DNase and 50 μg·mL^−1^ pancreatic RNase and was submitted to sonication (4 burst of 30 s, probe density 70). The suspension was then incubated for 2 h at 37 °C. Then, 0.5 M tetrasodium-EDTA, 100 μL of Tween 20 and 10 mM Tris-hydrochloride were added. The samples were centrifuged (10,000× *g*, 30 min, 20 °C) to remove the peptidoglycan. The supernatants were incubated overnight with 200 μg/mL of protease, at 37 °C, with constant shaking. Two volumes of 0.375 M MgCl_2_ in 96% ethanol were added. The samples were then centrifuged (12,000× *g*, 15 min, 4 °C), and the pellets were sonicated in a solution of Tween 20, 0.5 M tetrasodium-EDTA and 10 mM MgCl_2_. The pH of the solutions was lowered to 7 to prevent lipid saponification. The solutions were incubated for 30 min at 85 °C, to ensure that outer membrane proteins were denatured, and the pH of the solutions was increased to 9.5. Protease was then added, and the samples were incubated overnight at 37 °C. Two volumes of 0.375 M MgCl_2_ in 96% ethanol were added, and the samples were centrifuged (12,000× *g*, 15 min, 4 °C). The pellets were re-suspended in 10 mM Tris-HCl, sonicated and centrifuged twice to remove insoluble Mg^2+^-EDTA crystals. The supernatants were then centrifuged (62,000× *g*, 2 h, 15 °C). The pellets containing the LPS were re-suspended in distilled water. The LPS extracts were analyzed by Matrix-Assisted Laser Desorption/Ionization Time-of-Flight mass spectrometer (MALDI-TOF) using an Autoflex III TOF/TOF 200 MALDI mass spectrometer (Bruker Daltonics Wissembourg, France). For analysis, a 1 μL aliquot of purified LPS was spotted onto a steel target plate and air dried. A volume of 1 μL of a solution of α-cyano-4-hydroxycinnamic acid matrix (14 mg/mL in acetonitrile/2.5% trifluoroacetic acid, *v*/*v*) was added on each spot and dried at room temperature. The mass spectrometer was equipped with a pulsed YAG 200 Hz laser and was run in the positive mode. Instrument calibration was achieved by using calibration standards (Care, Bruker Daltonics, Wissembourg, France) spotted on the same target plate. Each spectrum was established over 200 laser shots.

### 4.10. Detection of Diffusible Virulence Factors

Little is known about diffusible virulence factors produced by *P. fluorescens*. In the present study, we focused on the production of exoenzymes and pyoverdine.

Secreted caseinase, esterase, amylase and hemolytic activities were studied from cultures on milk, Tween 80, starch and Columbia blood supplemented agar medium, respectively. For these tests, the medium was inoculated on the surface using a needle previously soaked with the centrifugation pellet and were incubated at 28 °C until the development of a halo revealing the bacterial enzymatic activity. The diameter of the halos was measured 24 h and 48 h after incubation.

The elastase activity was measured in liquid bacterial culture medium using an elastin/Congo red assay. Filtered supernatant (50 μL) was mixed with 1 mL of Tris buffer (0.1 M Tris-HCl pH 7.2, 1 mM CaCl_2_) containing 20 mg of elastin/Congo red (Sigma, St Quentin Fallavier, France). The tubes were incubated at 28 °C with agitation. After 18 h, the tubes were chilled on ice, and the reaction was stopped by adding 0.1 mL EDTA 0.12 M. Non-soluble elastin/Congo red was removed by centrifugation, and the OD_490_ was measured.

Pyoverdine production was monitored from 6 to 48 h of bacterial culture. To promote pyoverdine production, bacteria were grown on King B medium (for 1 L: 10 g glycerol; 20 g polypeptone; 1.5 g K_2_HPO_4_; 6 mM MgSO_4_, 7 H_2_O; pH 7) or in Bacto Casamino Acids (CAA) medium (for 1 L: 5 g of casamino acid; 0.9 g of K_2_HPO_4_; 2 mM MgSO_4_, 7H_2_O; pH 7.4). Pyoverdine production was expressed as the ratio OD_400_/OD_580_ of the supernatant after removal of the bacteria by centrifugation (5 min, 10,000× *g*).

### 4.11. Statistical Analysis

For all the results, each value reported for the assays is the mean of measurements from a minimum of three independent preparations. The Student *t*-test was used to compare the means within the same set of experiments.

## 5. Conclusions

In this present study, we demonstrate that GABA increases the cytotoxicity of *P. fluorescens* MF37 through modifications of the LPS structure. Furthermore, we observed that GABA can affect the biofilm formation activity and the surface polarity of the bacterium. These properties are not shared by all *P. fluorescens* strains, suggesting that GABA-sensitive strains adapted in response to specific environmental conditions. The mechanisms of virulence modulated by GABA in *P. fluorescens* and in *P. aeruginosa* are totally different, suggesting the existence of different detection and regulatory mechanisms.

## References

[1] Qi Z., Cort A. (2003). Free and combined amino compounds in atmospheric fine particles (PM_2.5_) and fog waters from Northern California. Atmos. Environ.

[2] Noe F.F., Nickerson W.J. (1958). Metabolism of 2-pyrrolidone and gamma aminobutyric acid by *Pseudomonas aeruginosa*. J. Bacteriol.

[3] Chou H.T., Kwon D.H., Hegazy M., Lu C.D. (2008). Transcriptome analysis of agmatine and putrescine catabolism in *Pseudomonas aeruginosa* PAO1. J. Bacteriol.

[4] Tunnicliff G. (1993). Inhibition of 4-aminobutyrate aminotransferase from *Pseudomonas fluorescens* by ATP. Biochem. Mol. Biol. Int.

[5] Kaspar H.F., Mountfort D.O., Pybus V. (1991). Degradation of gamma-aminobutyric acid (GABA) by marine microorganisms. FEMS Microbiol. Ecol.

[6] Mountfort D.O., Pybus V. (1992). Regulatory influences on the production of gamma-aminobutyric acid by a marine pseudomonad. Appl. Environ. Microbiol.

[7] Siragusa S., de Angelis M., di Cagno R., Rizzello C.G., Coda R., Gobbetti M. (2007). Synthesis of gamma-aminobutyric acid by lactic acid bacteria isolated from a variety of Italian cheeses. Appl. Environ. Microbiol.

[8] Richard H.T., Foster J.W. (2003). Acid resistance in *Escherichia coli*. Adv. Appl. Microbiol.

[9] Brechtel C.E., King S.C. (1998). 4-Aminobutyrate (GABA) transporters from the amine-polyamine-choline superfamily: Substrate specificity and ligand recognition profile of the 4-aminobutyrate permease from *Bacillus subtilis*. Biochem. J.

[10] Hu L.A., King S.C. (1998). Membrane topology of the *Escherichia coli* γ-aminobutyrate transporter: Implications on the topography and mechanism of prokaryotic and eukaryotic transporters from the APC superfamily. Biochem. J.

[11] Zhao Z., Ding J.Y., Ma W.H., Zhou N.Y., Liu S.J. (2012). Identification and characterization of gamma-aminobutyric acid uptake system GabP_Cg_ (NCgl0464) in *Corynebacterium glutamicum*. Appl. Environ. Microbiol.

[12] Planamente S., Mondy S., Hommais F., Vigouroux A., Moréra S., Faure D. (2012). Structural basis for selective GABA binding in bacterial pathogens. Mol. Microbiol.

[13] Dagorn A., Hillion M., Chapalain A., Lesouhaitier O., Duclairoir-Poc C., Vieillard J., Chevalier S., Taupin L., Le Derf F., Feuilloley M.G.J. (2013). Gamma-aminobutyric acid acts as a specific virulence regulator in *Pseudomonas aeruginosa*. Microbiology.

[14] Guthrie G.D., Nicholson-Guthrie C.S. (1989). Gamma-Aminobutyric acid uptake by a bacterial system with neurotransmitter binding characteristics. Proc. Natl. Acad. Sci. USA.

[15] Richard H., Foster J.W. (2004). *Escherichia coli* glutamate- and arginine-dependent acid resistance systems increase internal pH and reverse transmembrane potential. J. Bacteriol.

[16] Tramonti A., de Canio M., Delany I., Scarlato V., de Biase D. (2006). Mechanisms of transcription activation exerted by GadX and GadW at the gadA and gadBC gene promoters of the glutamate-based acid resistance system in *Escherichia coli*. J. Bacteriol.

[17] De Biase D., Pennacchietti E. (2012). Glutamate decarboxylase-dependent acid resistance in orally acquired bacteria: Function, distribution and biomedical implications of the gadBC operon. Mol. Microbiol.

[18] Chevrot R., Rosen R., Haudecoeur E., Cirou A., Shelp B.J., Ron E., Faure D. (2006). GABA controls the level of quorum-sensing signal in *Agrobacterium tumefaciens*. Proc. Natl. Acad. Sci. USA.

[19] Park D.H., Mirabella R., Bronstein P.A., Preston G.M., Haring M.A., Lim C.K., Collmer A., Schuurink R.C. (2010). Mutations in gamma-aminobutyric acid (GABA) transaminase genes in plants or *Pseudomonas syringae* reduce bacterial virulence. Plant J.

[20] Van de Mortel J.E., de Vos R.C., Dekkers E., Pineda A., Guillod L., Bouwmeester K., van Loon J.J., Dicke M., Raaijmakers J.M. (2012). Metabolic and transcriptomic changes induced in *Arabidopsis* by the rhizobacterium *Pseudomonas fluorescens* SS101. Plant Physiol.

[21] Chapalain A., Rossignol G., Lesouhaitier O., Merieau A., Gruffaz C., Guerillon J., Meyer J.M., Orange N., Feuilloley M.G.J. (2008). Comparative study of 7 fluorescent pseudomonad clinical isolates. Can. J. Microbiol.

[22] Mozrzymas J.W., Zarnowska E., Pytel M., Mercik K. (2003). Modulation of GABA_A_ receptors by hydrogen ions reveals synaptic GABA transient and a crucial role of the desensitization process. J. Neurosci.

[23] Shelp B.J., Bown A.W., Faure D. (2006). Extracellular gamma-aminobutyrate mediates communication between plants and other organisms. Plant Physiol.

[24] Johnson C.R., Muir D.G., Reysenbach A.L. (1991). Characteristic bacteria associated with surfaces of coralline algae: A hypothesis for bacterial induction of marine invertebrate larvae. Mar. Ecol. Prog. Ser.

[25] Picot L., Chevalier S., Mezghani-Abdelmoula S., Merieau A., Lesouhaitier O., Leroux P., Cazin L., Orange N., Feuilloley M.G.J. (2003). Cytotoxic effects of the lipopolysaccharide from *Pseudomonas fluorescens* on neurons and glial cells. Microb. Pathog.

[26] Angulo M.C., Le Meur K., Kozlov A.S., Charpak S., Audinat E. (2008). GABA, a forgotten gliotransmitter. Prog. Neurobiol.

[27] Picot L., Mezghani-Abdelmoula S., Chevalier S., Merieau A., Lesouhaitier O., Guerillon J., Cazin L., Orange N., Feuilloley M.G.J. (2004). Regulation of the cytotoxic effects of *Pseudomonas fluorescens* by growth temperature. Res. Microbiol.

[28] Bellon-Fontaine M.N., Rault J., van Oss C.J. (1996). Microbial adhesion to solvents: A novel method to determine the electron-donor/electron-acceptor or Lewis acid-base properties of microbial cells. Colloids Surf. B Biointerfaces.

[29] Cornelis P. (2010). Iron uptake and metabolism in pseudomonads. Appl. Microbiol. Biotechnol.

[30] Veron W., Lesouhaiter O., Pennanec X., Rehel K., Leroux P., Orange N., Feuilloley M.G.J. (2007). Natriuretic peptides affect *Pseudomonas aeruginosa* and specifically modify LPS biosynthesis. FEBS J.

[31] Owens D.F., Kriegstein A.R. (2002). Is there more to GABA than synaptic inhibition?. Nat. Rev. Neurosci.

[32] Bouché N., Lacombe B., Fromm H. (2003). GABA signaling: A conserved and ubiquitous mechanism. Trends Cell. Biol.

[33] Mezghani-Abdelmoula S., Khemiri A., Lesouhaitier O., Chevalier S., Orange N., Cazin L., Feuilloley M.G.J. (2004). Sequential activation of constitutive and inducible nitric oxide synthase (NOS) in rat cerebellar granule neurons by *Pseudomonas fluorescens* and invasive behaviour of the bacteria. Microbiol. Res.

[34] Fink S.L., Cookson B.T. (2005). Apoptosis, pyroptosis and necrosis: Mechanistic description of dead and dying eukaryotic cells. Infect. Immun.

[35] Veron W., Orange N., Feuilloley M.G.J., Lesouhaitier O. (2008). Natriuretic peptide modify *Pseudomonas fluorescens* cytotoxicity by regulating cyclic nucleotides and modifying LPS structure. BMC Microbiol.

[36] Guthrie G.D., Nicholson-Guthrie C.S., Leary H.L. (2000). A bacterial high-affinity GABA binding protein: Isolation and characterization. Biochem. Biophys. Res. Commun.

[37] Haas D., Defago G. (2005). Biological control of soil-borne pathogens by fluorescent pseudomonads. Nat. Rev. Microbiol.

[38] Bown A.W., MacGregor K.B., Shelp B.J. (2006). Gamma-aminobutyrate: Defense against invertebrate pests?. Trends Plant Sci.

[39] Kumar P., Kalonia H., Kumar A. (2012). Possible GABAergic mechanism in the neuroprotective effect of gabapentin and lamotrigine against 3-nitropropionic acid induced neurotoxicity. Eur. J. Pharmacol.

[40] Davey M.E., O’Toole G.A. (2000). Microbial biofilms: From ecology to molecular genetics. Microbiol. Mol. Biol. Rev.

[41] Ito K., Tanaka K., Nishibe Y., Hasegawa J., Ueno H. (2007). GABA-synthesizing enzyme, GAD67, from dermal fibroblasts: Evidence for a new skin function. Biochim. Biophys. Acta.

[42] Grice E.A., Kong H.H., Renaud G., Young A.C., Bouffard G.G., Blakesley R.W., Wolfsberg T.G., Turner M.L., Segre J.A. (2008). A diversity profile of the human skin microbiota. Genome Res.

[43] Gao Z., Tseng C., Pei Z., Blaser M.J. (2007). Molecular analysis of human forearm superfical skin bacterial biota. Proc. Natl. Acad. Sci. USA.

[44] Raaijmakers J.M., De Bruijn I., Nybroe O., Ongena M. (2010). Natural functions of lipopeptides from *Bacillus* and *Pseudomonas*: More than surfactants and antibiotics. FEMS Microbiol. Rev.

[45] Becerra C., Albesa I., Eraso A.J. (2001). Leukotoxicity of pyoverdin, production of reactive oxygen species, and effect of UV radiation. Biochem. Biophys. Res. Commun.

[46] Makin S.A., Beveridge T.J. (1996). The influence of A-band and B-band lipopolysaccharide on the surface characteristics and adhesion of *Pseudomonas aeruginosa* to surfaces. Microbiology.

[47] Yokota S., Fujii N. (2007). Contributions of the lipopolysaccharide outer core oligosaccharide region on the cell surface properties of *Pseudomonas aeruginosa*. Comp. Immunol. Microbiol. Infect. Dis.

[48] Mitrovic B., Ignarro L.J., Vinters H.V., Akers M.-A., Schmid I., Uittenbogaart C., Merrill J.E. (1995). Nitric oxide induces necrotic but not apoptotic cell death in oligodendrocytes. Neuroscience.

[49] Burini J.F., Gügi B., Merieau A., Guespin-Michel J.F. (1994). Lipase and acidic phosphatase from the psychrotrophic bacterium *Pseudomonas fluorescens*: Two enzymes whose synthesis is regulated by the growth temperature. FEMS Microbiol. Lett.

[50] Silby M.W., Cerdeño-Tárraga A.M., Vernikos G.S., Giddens S.R., Jackson R.W., Preston G.M., Zhang X.X., Moon C.D., Gehrig S.M., Godfrey S.A. (2009). Genomic and genetic analyses of diversity and plant interactions of *Pseudomonas fluorescens*. Genome Biol.

[51] O’Toole G.A., Kolter R. (1998). Flagellar and twitching motility are necessary for *Pseudomonas aeruginosa* biofilm development. Mol. Microbiol.

[52] Bazire A., Diab F., Jebbar M., Haras D. (2007). Influence of high salinity on biofilm formation by *Pseudomonas aeruginosa*. J. Ind. Microbiol. Biotechnol.

[53] Hiemenz P.C., Lagowski J.J., Lagowski J.J. (1977). Principles of Colloid and Surface Chemistry.

[54] Darveau R.P., Hancock R.E. (1983). Procedure for isolation of bacterial lipopolysaccharides from both smooth and rough *Pseudomonas aeruginosa* and Salmonella typhimurium strains. J. Bacteriol.

